# The Nightingale Legend: The Reaction and the Reality

**Published:** 1918-10-19

**Authors:** 


### THE NIGHTINGALE LEGEND.

### The Reaction and the Reality.

MR. LYTTON STRACHEY, whose "Eminent Victorians" (published by Messrs. Chatto and Windus, 10s. 6d. net) has caused so much amusing consternation among many successful and earnest persons who will be none the worse for his delicate disturbance of their complacency, is, on his own scale, the Twentieth Century Macaulay. Just as that lively writer represented the bumptiousness of the nineteenth century in its nonage, and enchanted those who were unequal to the strain of Johnson and Gibbon by the rhetoric and vivid portraiture of his essays, and by the so-called history, which he made at once more exciting than the current novel; and whose substance he reduced to the level of a sham party crisis in the House; so Mr. Strachey represents the ironic assumption of indifference which is characteristic of our modern youth, which mostly falls between the stools of an ideal in the tradition of which the young no longer live, and the reality which they have not yet discovered. After Ibsen, Macaulay's optimism is found a little vulgar; as for *blasé* pessimism, it always was, since cheerfulness is an important part of good manners. To those who have no philosophy, irony alone remains if they are intellectually alert people, and it is particularly congenial to the atmosphere of King's College, Cambridge, where there is a leaven of Eton, a youthful and healthy worship of the Comic Spirit, and, among the 1903-14 undergraduates, such culture as omnivorous reading, mostly in the paddock of modern literature, can give.

Clarifying is Mr. Strachey's province, a province which, among the younger generation of historical essayists, he has made his own. Note, too, that these things he sees are those which most men see who see nothing else; that they are indeed the preoccupation of party leaders, social climbers, and the ubiquitous "getter-on" in shop and office, City and club, Holy Orders, and all the professions. To see the proportions of all this is child's play to Mr. Strachey; for he is intellectually alive to every ripple of metropolitan and university life; and, in consequence, takes a keen pleasure in flashing the limelight upon now this and now that aspect of the crowded panorama. There is sufficient of the dramatist in him to see every man in this crowd, if not from the man's own point of view, at least from that of his friend and neighbour, provided that either point of view is, essentially, that of the shrewd, observant average mind. This proviso must be added, for there be some that are in the crowd who are yet not of it: seers, dreamers, artists, founders of religions, mystics, prophets, poets! To these he has not the divinatory clue; he has not in him a share of that quality by which, as by a secret sign, they recognise each other in a moment. To that which is Reality to them he is blind. It is not within his compass; for he is an observer rather than a thinker, a writer of extraordinary talent, not a creator, an ironist not that rare bird, a satirist, a looker-on at the human comedy, not a protagonist in the great adventure of life. Therefore, in considering his representations of the four "Eminent Victorians" who are the subjects of his essays, we must remember that each had some degree of genius, and that in so far as this entered into their personality and lives, it queers the pitch for Mr. Strachey, makes him, for the moment, lose his air of detachment and become, at meteoric moments, a figure of comedy. As Mrs. Humphry Ward said in a letter of self-betrayal, every *nuance* in the flavour of which must have been exquisitely apparent to Mr. Strachey: in an estimate of a character irony does not say the final word: how much it does say, perhaps, she is unable, in the circumstances, to admit. The truth of her contention is really contained in the motto which the author has chosen for his book: the phrase which he quotes in justification at once of his method and of his mood; the phrase "J'expose." Yes, but the emphasis is even more upon the pronoun, and an attentive reader, after enjoying the last, suppressed smile at the expense of their Eminences, will find that he knows much about Mr. Strachey, and also knows perhaps more than Mr. Strachey himself about a certain quality common to the Four.

The popular conception of Florence Nightingale offered an irresistible subject to the author, and he is careful, of course, to devote the first paragraph of his essay to its delineation. The "saintly, self-sacrificing woman of high degree who threw aside the pleasures of a life of ease to succour the afflicted," and to be rewarded by popular canonisation as the Lady with the Lamp, is, as he adds, familiar to us all. "The truth was different," he concludes. "A Demon possessed her . . . and it so happens that in the real Miss Nightingale there was more that was interesting than in the legendary one; there was also less that was agreeable." This legend, however, is already dead, though the writer of "Eminent Victorians" may seem at first to be only the reaction against it. It was replaced through the publication of Sir E. T. Cook's monumental biography, the one standard Life which Mr. Strachey feels able to describe as "an honourable exception" to the tedious and truth-disguising rule. Sir E. T. Cook's work is indeed an exception to that. It is the best example of the current conception of biography.

If our attempt to present the process of his mind be correct, we shall hardly be surprised that the moment which is emphasised in his picture is the death of Sidney Herbert, which he does not hesitate to attribute to the overwork thrust upon him by Miss Nightingale. Her Demon rather than herself is held responsible. The next point which arrests his eye is the inscription on the brooch designed by the Prince Consort and presented to

**The Nightingale Legend**—(*continued*).

her on her return from the Crimea by Queen Victoria. The words ran: "Blessed are the merciful." Sidney Herbert's hour had not yet come.

A greater Alexander, however, Miss Nightingale never supposed that the time would ever come for her when there would be no more worlds to conquer. The vast sphere of practical work, in begetting the germ of the modern hospital, in establishing nursing as an honourable profession for educated women, in sanitary reform of all kinds, in improving the existing Poor Law, in the "passionate" collection of statistics: this was not enough. She began to meditate upon disease, and evolved a very remarkable, if succinct, philosophy upon it. The study of the philosophy of disease led her to study its origin. There remained behind even that, the problem of the nature of God. Her strange and varied friendships included intimacies with John Stuart Mill, and Dr. Jowett of Balliol. She devoured their works; she even revised them at the authors' instance. The time when she herself would write was now anticipated in her own mind. Had the latest word been said? She knew, in spite of Mr. Strachey's hesitation on this point, that it would not be the last one (for *she* had a sense of humour). Could not she begin where Plato had left off? Did even Jowett fully understand? Her Demon urged her, as Mr. Strachey would put it, to have a finger in this pie also. After Plato, the mystics. What had these to teach on the intertissued subject of the nature of God and the scientific solution of drainage? She was sufficiently intelligent to see that all necessary problems are related; and sufficiently a poet to see, too, that the principles of philosophy and those of science are, or can be, one. After the principles, necessarily came their application. After the philosophy of disease, a system of treatment, the hospital problem, the training of medical students and nurses. After the divination of our relation to God, the rules of conduct and of manners. On the elucidation of the principles therefore, encouraged by the speculative minds of Jowett and Mill, she set to work. An early work, still the classic on its subject, called "Notes on Nursing," showed her to possess a fine, nervous style, a grasp of principles, a sense of proportion, an alert, observant eye, and a pungent wit. The instrument was there. Had she not once indulged, and then rejected, literary ambitions? The gift, at the Demon's call, should now be put to vital issues. Her "Suggestions for Thought" came to be written. Here Mr. Strachey is, though far from self-confessedly, puzzled. That he is puzzled seems to be evident, however, from the following passage (p. 171).

"A law," she had pointed out, "implies a law-giver." Now the universe is full of laws; . . . hence it follows that the universe has a law-giver—and what would Mr. Mill be satisfied with, if he was not satisfied with that? Perhaps Mr. Mill might have asked why the argument should not be pushed to its logical conclusion. Clearly, if we are to trust the analogy of human institutions, we must remember that laws are, as a matter of fact, not dispensed by law-givers, but passed by Act of Parliament. Miss Nightingale, however, with all her experience of public life, never stopped to consider whether God might not be a Limited Monarchy.

Mr. Strachey here stumbles over the shins of his own wit. His phrase indeed might be hers. Had he studied Florence Nightingale's "Suggestions" more attentively, and properly related them to her views on statistics, he would have found that the former, especially, postulated the existence of God, and that the latter held God to be limited by the laws of His own universe: notwithstanding the existence of the Divine Prerogative, which, like the Royal Prerogative of a limited monarchy, is precisely the element which reigns but does not rule, and retains its virtue best when invoked on extraordinary occasions only. This humorous oversight, which leaves us the irony and disarms the thrust which Mr. Strachey fancied to be so fatal, disables the IVth section of the essay, and indicates the element of understanding lacking here and there throughout the whole. There is a blind spot in Mr. Strachey's critical retina. The final clue to great characters is wanting in him. He has little comprehension of the experience which goes to the making of the poet, the mystic, and the seer, that experience which lies at the root of mysticism, and enables us to define it, with perhaps sufficient conciseness, as a first-hand experience of Reality. It is this lack, this want of apprehension, as Patmore understood the word, which leads the essayist to the hasty conclusion that "the scientific method itself was alien to" Miss Nightingale's "spirit," and to the still wilder statement that she was simply an "empiricist." The truth is that she had an intuitive apprehension of first principles, but that, living in an age of "diffidence," she was far from invariably right in her application of them. Believing in open-air wards (as he notices), she wished to enforce them in all conditions and climates, without regard to the other less desirable things which in India, say, are understood to enter through perpetually open windows. The principle was right, the application should have been proportional. A mere empiric, *pace* Mr. Strachey, would have ordered all windows to be shut, because a sick person especially dreads, and is in danger from, draughts. The philosophical mind, that which Matthew Arnold finely called "imaginative reason," avoids little blunders of this kind, and in consequence is not puzzled by Miss Nightingale. It recognises the real type to which she belongs by the pronounced presence in her of the mark which is invariably diagnostic of it. The type is that of the great mystic. The great mystic is known by a combination of qualities to be found in no other class of human beings—the combination, namely, of enormous practical and administrative ability with intense visionary or apprehensive power. In the St. Catharines (of Siena) and the St. Teresas (of Spain) the two qualities are apparently in equipoise, though, at the

(*Concluded on p.* 48.)

### The Nightingale Legend.

(*Concluded from page 50.*)

height to which they attain in this ocean brood, an unstable equipoise no doubt. The type at this stage of development is rare in modern times, because, perhaps, the conditions of the modern age are more than usually opposed to its development. Consequently in Florence Nightingale we find, as we should expect to find, the organising quality in excess of the apprehensive; her acts liable to prove more uniform and in harmony than her apprehensions, Suggestions, and ideas. Mr. Strachey has given us something: he has "potted" the big biography so prettily, that it would be idle to lament that he has not given us all. The omission would not have been worth mentioning, were not a due emphasis upon its existence in her, with all the rest that Mr. Strachey's keen eye collates in order, necessary, indeed vitally necessary, to an adequate appreciation of the profile presented by her personality.

